# [^18^F]FDG PET/MRI combined with chest HRCT in early cancer detection: a retrospective study of 3020 asymptomatic subjects

**DOI:** 10.1007/s00259-023-06273-6

**Published:** 2023-07-04

**Authors:** Liling Peng, Yi Liao, Rui Zhou, Yan Zhong, Han Jiang, Jing Wang, Yu Fu, Le Xue, Xiaohui Zhang, Mingxiang Sun, Gang Feng, Zhaoting Meng, Sisi Peng, Xuexin He, Gaojun Teng, Xin Gao, Hong Zhang, Mei Tian

**Affiliations:** 1 Shanghai Universal Medical Imaging Diagnostic Center, Shanghai, China; 2 Department of Nuclear Medicine and PET-CT Center, The Second Affiliated Hospital of Zhejiang University School of Medicine, Zhejiang, Hangzhou China; 3 Key Laboratory of Medical Molecular Imaging of Zhejiang Province, Hangzhou, China; 4 Institute of Nuclear Medicine and Molecular Imaging of Zhejiang University, Hangzhou, China; 5 College of Information Science & Electronic Engineering, Zhejiang University, Hangzhou, China; 6 Human Phenome Institute, Fudan University, Shanghai, China; 7 Department of Oncology, Huashan Hospital, Fudan University, Shanghai, China; 8 Radiology Department, Zhongda Hospital Southeast University, Nanjing, Jiangsu China; 9 Key Laboratory for Biomedical Engineering of Ministry of Education, Zhejiang University, Zhejiang, Hangzhou China; 10 The College of Biomedical Engineering and Instrument Science, Zhejiang University, Zhejiang, Hangzhou China

**Keywords:** Positron emission tomography/magnetic resonance imaging (PET/MRI), Glucose metabolism, Early detection of cancer

## Abstract

**Purpose:**

PET/MRI has become an important medical imaging approach in clinical practice. In this study, we retrospectively investigated the detectability of fluorine-18 (^18^F)-fluorodeoxyglucose positron emission tomography/magnetic resonance imaging ([^18^F]FDG PET/MRI) combined with chest computerized tomography (CT) for early cancer in a large cohort of asymptomatic subjects.

**Methods:**

This study included a total of 3020 asymptomatic subjects who underwent whole-body [^18^F]FDG PET/MRI and chest HRCT examinations. All subjects received a 2–4-year follow-up for cancer development. Cancer detection rate, sensitivity, specificity, positive predictive value (PPV), and negative predictive value (NPV) of the [^18^F]FDG PET/MRI with or without chest HRCT were calculated and analyzed.

**Results:**

Sixty-one subjects were pathologically diagnosed with cancers, among which 59 were correctly detected by [^18^F]FDG PET/MRI combined with chest HRCT. Of the 59 patients (32 with lung cancer, 9 with breast cancer, 6 with thyroid cancer, 5 with colon cancer, 3 with renal cancer, 1 with prostate cancer, 1 with gastric cancer, 1 with endometrial cancer, and 1 with lymphoma), 54 (91.5%) were at stage 0 or stage I (according to the 8th edition of the tumor-node-metastasis [TNM] staging system), 33 (55.9%) were detected by PET/MRI alone (27 with non-lung cancers and 6 with lung cancer). Cancer detection rate, sensitivity, specificity, PPV, and NPV for PET/MRI combined with chest CT were 2.0%, 96.7%, 99.6%, 83.1%, and 99.9%, respectively. For PET/MRI alone, the metrics were 1.1%, 54.1%, 99.6%, 73.3%, and 99.1%, respectively, and for PET/MRI in non-lung cancers, the metrics were 0.9%, 93.1%, 99.6%, 69.2%, and 99.9%, respectively.

**Conclusions:**

[^18^F]FDG PET/MRI holds great promise for the early detection of non-lung cancers, while it seems insufficient for detecting early-stage lung cancers. Chest HRCT can be complementary to whole-body PET/MRI for early cancer detection.

**Trial registration:**

ChiCTR2200060041. Registered 16 May 2022. Public site: https://www.chictr.org.cn/index.html

**Supplementary Information:**

The online version contains supplementary material available at 10.1007/s00259-023-06273-6.

## Introduction

Positron emission tomography/magnetic resonance imaging (PET/MRI) is a hybrid imaging technique that has been used in clinical practice. Compared with positron emission tomography/computed tomography (PET/CT), PET/MRI provides reduced radiation exposure and enhanced morphological soft-tissue contrast. Therefore, PET/MRI has the potential to improve diagnostic evaluation by providing complementary molecular and anatomical information [1].

Cancer is one of the leading causes of death and threatens to shorten human life expectancy worldwide [2]. Patients with advanced cancers often indicate a poor prognosis and heavy economic burden [3]. The development of PET/CT greatly improves the detection rate and management of cancers [4]. Recently, the introduction of PET/MRI, another hybrid imaging technique, has been utilized in the diagnosis of neurological diseases, soft tissue sarcoma, primary prostate cancer, and pediatric cancers [5]. The value of fluorine-18 (^18^F)-fluorodeoxyglucose (FDG) PET/MRI in cancer management has been evaluated in several cancers, including esophageal cancer, breast cancer, and pediatric tumors [6–8]. These studies involved in primary cancer detection, cancer metastasis, TNM staging, and prognosis of clinically significant cancer patients. However, this indicated that most patients were diagnosed at advanced stages, which means these patients might lose the chance to early intervene the cancer progression. A number of patients with cancer (about 70–80%) died due to the delayed diagnosis of primary and metastasis lesions [9, 10]. Therefore, early cancer detection could bring benefits to the survival of cancer patients.

Hybrid imaging techniques possess the ability to provide a wealth of information encompassing anatomical, functional, and molecular information, which could give a whole-body readout in an intact system and aid in early cancer detection [11]. As a hybrid imaging technique, [^18^F]FDG PET/MRI inherits the high sensitivity of PET as well as the high anatomic resolution of MRI. Thus, PET/MRI has the potential to detect unexpected cancers in different organs by surveying the entire body in a single examination with noninvasive and painless procedures. However, the ability of PET/MRI in detecting pulmonary nodules is poorer than high-resolution CT (HRCT) [12]. Compared with PET/MRI, HRCT could detect small pulmonary nodules and provide more anatomical information, which is essential for differential diagnosis of ground-grass nodule (GGO) [13]. Therefore, PET/MRI combined with HRCT might improve the diagnosis efficiency to screen the whole body for cancer detection.

Though PET/MRI has been widely admitted to oncological imaging, the value of [^18^F]FDG PET/MRI for whole-body cancer screening is still unclear. Currently, some studies have been conducted on the diagnostic values of PET/MRI for local regions of the human body [14, 15]; it is still lack of research involved in investigating PET/MRI as a general cancer detection modality for the whole body, especially based on a large population. Meanwhile, HRCT might make up the defect of [^18^F]FDG PET/MRI in detecting pulmonary lesions. Thus, this study aimed to investigate the detectability of whole-body PET/MRI combined with HRCT in early cancer detection in a large population of asymptomatic subjects.

## Materials and methods

### Study subjects and design

**Fig. 1:**
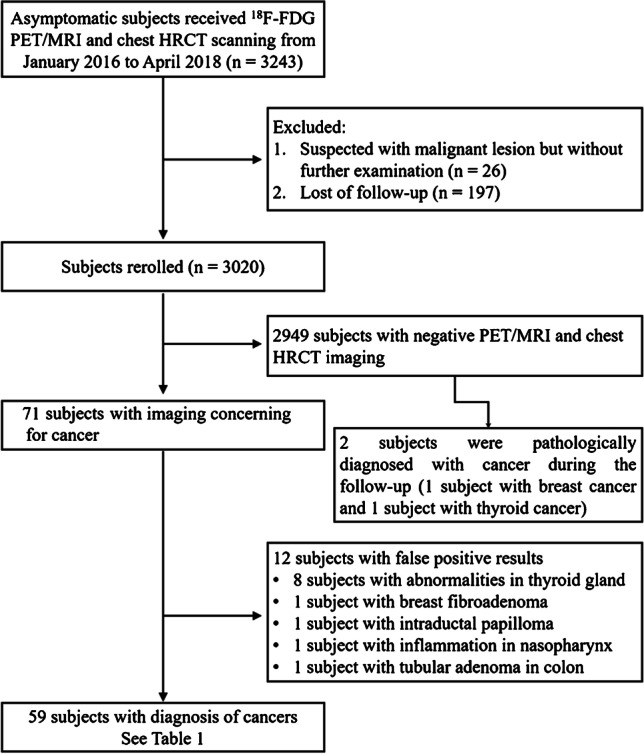
Flowchart describing the study of [^18^F]FDG PET/MRI combined with chest HRCT. The diagnostic path indicates how cancers in asymptomatic subjects were detected and identified

We retrospectively enrolled 3243 asymptomatic subjects between January 2016 and April 2018 in Universal Imaging Diagnostic Center (Shanghai, China). The inclusion criteria were as follows: (1) who are willing and afford to conduct whole-body cancer screening; (2) over 18 years old; (3) underwent both [^18^F]FDG PET/MRI and chest HRCT; (4) with negative cancer history, precancerous lesions, clinical symptoms of cancers, history of hepatitis B, HPV infection, or abnormal serum tumor markers. Subjects were excluded if they (1) were suspected with malignant lesion but without further examination and (2) were lost to follow-up. The exclusion criteria for [^18^F]FDG PET/MRI imaging were as follows: (1) contraindications to MRI scanning, including incompatible metallic hardware or devices, ocular metallic foreign bodies, and claustrophobia, (2) pregnancy, (3) blood glucose levels over 140 mg/dL (7.77 mmol/L), and (4) poor PET/MRI image quality, due to artifacts, system malfunction, or poor cooperation. Finally, 3020 asymptomatic subjects were enrolled in our study. The study was approved by the Shanghai Ethics Committee for Clinical Research (Approval Number: SECCR/2021-125-01). The flowchart of subject enrollment is shown in Fig. 1.

### Imaging protocols

CT images without contrast enhancement of the chest (Somatom Definition Flash, Siemens Healthcare, 120 kV, auto-mAs mode, pitch of 1.2, reconstructed to 1-mm slices) were obtained before PET/MRI scans.

[^18^F]FDG PET/MRI images were acquired and reconstructed by using 3.0T PET/MRI device (Biograph mMR, Siemens Healthcare, Erlangen, Germany). The version of the PET/MRI operating system was syngo MRI VB20P (Siemens Healthcare GmbH). All subjects fasted for at least 6 h and serum glucose levels were confirmed prior to the FDG injection. The scanning field was from the midthigh level to the top of head with the subject in the supine position. Subjects received intravenous administration of [^18^F]FDG (3.7MBq/kg). After 45-min rest in dim and quiet environment, a whole-body PET scan was performed with a three-dimensional volumetric interpolated breath-hold examination Dixon sequence for MRI attenuation correction (Dixon-based four-segment μ map) for 3–4 min per bed position in 5–6 beds following by the fast-view T1-weighted MRI localizer sequence for scout imaging. The PET and MRI scans were started at the same table position and time, thus ensuring optimal temporal and spatial correspondence between MRI and PET images. Each subject underwent the same basic sequences. An additional pelvic T2WI sequence in sagittal view was added for female. The MRI sequences and technical details included in the PET/MRI imaging protocol are introduced in Table S1. PET images were reconstructed using the ordinary poisson ordered subset expectation maximization (OSEM), with three iterations, 21 subsets, and a 5-mm Gaussian post processing filter, into 172 × 172 matrices. The average time for a whole-body PET/MRI examination was 40–45 min.

### Image analysis

[^18^F]FDG PET/MRI and chest HRCT images were evaluated at a dedicated workstation (Syngo.via; Siemens Healthcare) by senior board-certified nuclear medicine physicians including 3 independent readers. Nineteen organs/tissues (brain, thyroid, breast, esophagus/stomach, lung, liver, spleen, pancreas, small bowel/large bowel, uterus/cervix/ovaries, kidney, adrenal gland, lymph node, bone, and soft tissues) were mainly included to estimate whether there are positive malignant lesions, based on FDG uptake and/or morphological CT/MRI features. Briefly, PET, MRI, and CT images were evaluated separately, and one of the following results was obtained for each item: suggestive of malignant tumors, possible of malignant tumors, no evidence of benign or malignant tumor. Only if one of the three images was evaluated as suggestive of malignant tumors, the result was considered suggestive of malignant tumors. Any combination of one or two possible of malignant tumors with no evidence of benign or malignant tumors is classified as possible of malignant tumors. Both suggestive and possible of malignant tumors were counted as a positive result, and further diagnosis and treatment were recommended. The diagnosis standard of PET is increased abnormal [^18^F]FDG uptake, and the diagnosis of MRI and CT mainly depended on the morphology of the tumor and the change of MRI signal. The inconsistent cases were reviewed and adjudicated by the two readers together to reach a consensus.

When abnormalities were found (suggestive or possible of malignant tumors), subjects were referred to other hospitals for histological examination and definitive diagnosis; then, the pathological reports were obtained from subjects by contacts. The follow-up was performed after the completion of scanning and ended in October 2020. All subjects were followed up for 2–4 years to ascertain the occurrence of cancers. The endpoints were set at the cancer ascertainment. Subjects were classified as normal or benign, if they lacked evidence of malignancy during the follow-up.

### Statistical analysis

SPSS Statistics, version 22.0 (SPSS Inc.), was used to perform statistical analysis. A positive detecting result associated with a cancer diagnosis during the follow-up was defined as a true positive (TP) (or false positive (FP) if not associated with cancer diagnosis). A negative result without cancer development during the follow-up was defined as a true negative (TN) (or false negative (FN) if with cancer development). Cancer detection rate, sensitivity, specificity, positive predictive value (PPV), negative predictive value (NPV), and their respective 95% confidence intervals were calculated to estimate the detecting performance. These metrics were calculated as follows: sensitivity = TP/(TP+FN), specificity = TN/(TN+FP), PPV = TP/(TP+FP), and NPV = TN/(TN+FN). McNemar’s test was employed to compare the diagnostic performance of [^18^F]FDG PET/MRI and chest HRCT.

## Results

### Subject characteristics

Between January 2016 and April 2018, 3020 asymptomatic subjects were enrolled after excluding 197 subjects losing contact and 26 subjects suspected with malignant lesion but without further examination. There are 1938 male and 1082 female including in these subjects, in which male to female ratio is 1.79:1. The average age of subjects was 49.7 ± 9.7 years (ranging from 20 to 86 years). The majority of the population comprised subjects in their 40s and 50s, accounting for 70.7%. Details of characteristics of the 3020 subjects are listed in Table 1.

**Table 1:** Characteristics of the subjects

Characteristic	Male	Female	Total
Population (number %)	1938 (64.2%)	1082 (35.8)	3020 (100%)
Mean age (years)	49.6 ± 9.3	49.7 ± 10.3	49.7 ± 9.7
Age groups (number %)			
20–29	20 (0.7%)	28 (0.9%)	48 (1.6%)
30–39	230 (7.6%)	149 (4.9%)	379 (12.5%)
40–49	722 (23.9%)	360 (11.9%)	1082 (35.8%)
50–59	697 (23.1%)	357 (11.8%)	1054 (34.9%)
60–69	233 (7.7%)	153 (5.1%)	386 (12.8%)
70–79	31 (1.0%)	29 (1.0%)	60 (2.0%)
80–89	5 (0.2%)	6 (0.2%)	11 (0.4%)
Mean weight (kg)	74.4 ± 11.9	58.6 ± 8.5	68.7 ± 13.2
Mean height (cm)	170.8 ± 14.2	160.2 ± 7.3	167.0 ± 13.2
Mean BMI (kg/m^2^)	25.3 ± 3.1	22.8 ± 3.1	24.4 ± 3.31

*BMI* body mass index

### Lesion detection by [^18^F]FDG PET/MRI and chest HRCT

According to the standard of reference, a total of 71 subjects were considered to be suggestive of malignancy based on whole-body [^18^F]FDG PET/MRI and chest HRCT cancer detection, of which 59 (25 male and 34 female) were correctly diagnosed and pathologically confirmed with cancers (Table 2). Wherein, thirty-nine patients (66.1%) were detected at the ages of 40s and 50s, which is corresponding to the age distribution.

**Table 2:** Information of malignant tumors detected by whole-body PET/MR and chest CT

No.	Sex	Age	Classification	PET/MRI		CT	Pathologic diagnosis	TNM stage	Location	Size (cm)
				PET	MRI					
1	F	46	S	S	P	-	Endometrial cancer	Stage I	Endometrium	0.7×0.5
2	M	53	S	S	S	-	Gastric cancer	Stage II	Gastric body	4.1×1.2
3	M	65	S	N	S	-	Renal carcinoma	Stage I	Left kidney	1.6×1.3
4	F	50	S	N	S	-	Renal carcinoma	Stage I	Right kidney	4.5×3.6
5	M	38	S	N	S	-	Renal carcinoma	Stage I	Left kidney	0.8×0.7
6	F	57	S	S	S	N	Breast cancer	Stage I	Right breast	1.3×0.6
7	F	66	S	S	S	N	Breast cancer	Stage I	Right breast	1.7×1.2
8	F	61	P	P	P	N	Breast cancer	Stage I	Left breast	2.0×0.8
9	F	60	S	S	P	N	Breast cancer	Stage I	Left breast	1.7×0.7
10	F	58	S	S	S	P	Breast cancer	Stage III	Left breast	7.0×2.9
11	F	52	S	S	S	N	Breast cancer	Stage I	Left breast	1.2×1.0
12	F	49	S	S	S	N	Breast cancer	Stage I	Right breast	1.3×1.0
13	F	47	S	S	S	N	Breast cancer	Stage III	Right breast	4.1×2.1
14	F	37	S	S	S	N	Breast cancer	Stage I	Left breast	1.1×1.0
15	M	43	P	P	P	P	lymphoma	Stage I	Anterior mediastinum	3.3×1.7
16	M	63	S	S	S	-	Prostate cancer	Stage I	Left peripheral zone	1.8×1.4
17	M	53	P	P	P	-	Colon cancer	Stage I	Sigmoid colon	1.9×1.3
18	M	77	P	P	P	-	Colon cancer	Stage I	Ascending colon	2.1×1.9
19	F	64	P	P	P	-	Colon cancer	Stage I	Sigmoid colon	2.3×1.9
20	M	56	P	P	P	-	Colon cancer	Stage I	Descending colon	2.6×2.0
21	F	52	S	S	S	-	Colon cancer	Stage III	Ascending colon	3.9×3.4
22	M	56	P	P	P	N	Thyroid cancer	Stage I	Right lobe	0.4×0.4
23	M	50	S	S	P	N	Thyroid cancer	Stage I	Right lobe	0.8×0.7
24	F	62	S	S	P	N	Thyroid cancer	Stage I	Left lobe	1.0×0.8
25	M	55	S	S	S	S	Thyroid cancer	Stage II	Right lobe	1.3×1.3
26	M	54	P	P	P	N	Thyroid cancer	Stage I	Right lobe	1.2×1.1
27	F	53	S	S	P	N	Thyroid cancer	Stage I	Left lobe	0.7×0.3
28	F	51	S	N	N	S	Lung cancer	Stage 0	Left lung	0.6×0.4
29	M	80	S	S	S	S	Lung cancer	Stage I	Left lung	1.7×1.1
30	F	84	S	N	N	S	Lung cancer	Stage 0	Right lung	1.0×0.9
31	F	68	S	N	N	S	Lung cancer	Stage I	Right lung	1.0×0.6
32	F	64	S	N	N	S	Lung cancer	Stage I	Left lung	1.0×0.7
33	F	63	S	S	S	S	Lung cancer	Stage I	Right lung	2.4×0.7
34	M	61	P	N	N	P	Lung cancer	Stage I	Right lung	1.9×0.8
35	F	59	S	P	P	S	Lung cancer	Stage I	Right lung	0.8×0.8
36	F	57	P	P	P	P	Lung cancer	Stage I	Right lung	1.1×0.6
37	M	53	P	N	N	P	Lung cancer	Stage I	Right lung	0.6×0.6
38	F	52	S	N	N	S	Lung cancer	Stage 0	Left lung	0.6×0.5
39	M	52	S	N	N	S	Lung cancer	Stage 0	Right lung	0.8×0.7
40	M	51	S	N	N	S	Lung cancer	Stage 0	Right lung	1.0×0.8
41	F	51	S	N	N	S	Lung cancer	Stage 0	Left lung	0.6×0.5
42	M	51	P	N	N	P	Lung cancer	Stage I	Right lung	0.8×0.5
43	M	51	S	N	N	S	Lung cancer	Stage I	Left lung	1.6×1.0
44	F	48	S	N	N	S	Lung cancer	Stage 0	Right lung	1.0×0.8
45	F	48	S	N	N	S	Lung cancer	Stage I	Right lung	0.7×0.5
46	F	46	S	N	N	S	Lung cancer	Stage 0	Right lung	0.6×0.5
47	M	46	S	P	P	S	Lung cancer	Stage I	Right lung	2.9×1.8
48	F	46	S	N	N	S	Lung cancer	Stage I	Left lung	0.5×0.4
49	M	46	S	N	N	S	Lung cancer	Stage I	Left lung	0.6×0.4
50	M	46	S	N	N	S	Lung cancer	Stage I	Right lung	0.8×0.5
51	F	45	S	N	N	S	Lung cancer	Stage 0	Right lung	1.1×0.8
52	M	45	S	N	N	S	Lung cancer	Stage I	Right lung	3.0×1.7
53	F	44	S	N	N	S	Lung cancer	Stage 0	Right lung	0.6×0.4
54	M	44	S	N	N	S	Lung cancer	Stage I	Left lung	1.6×0.9
55	F	41	S	N	N	S	Lung cancer	Stage I	Right lung	0.8×0.5
56	M	39	S	N	N	S	Lung cancer	Stage 0	Right lung	0.6×0.5
57	F	38	S	N	N	S	Lung cancer	Stage 0	Right lung	0.6×0.5
58	F	34	S	P	P	S	Lung cancer	Stage I	Right lung	1.4×1.2
59	F	30	S	N	N	S	Lung cancer	Stage I	Left lung	0.8×0.5

*F* female, *M* male, *S* suggestive of malignancy, *P* possible of malignancy, *N* no evidence of benign or malignant tumor, -represent no CT scan of the tumor located organ/tissue was carried out

**Fig. 2:**
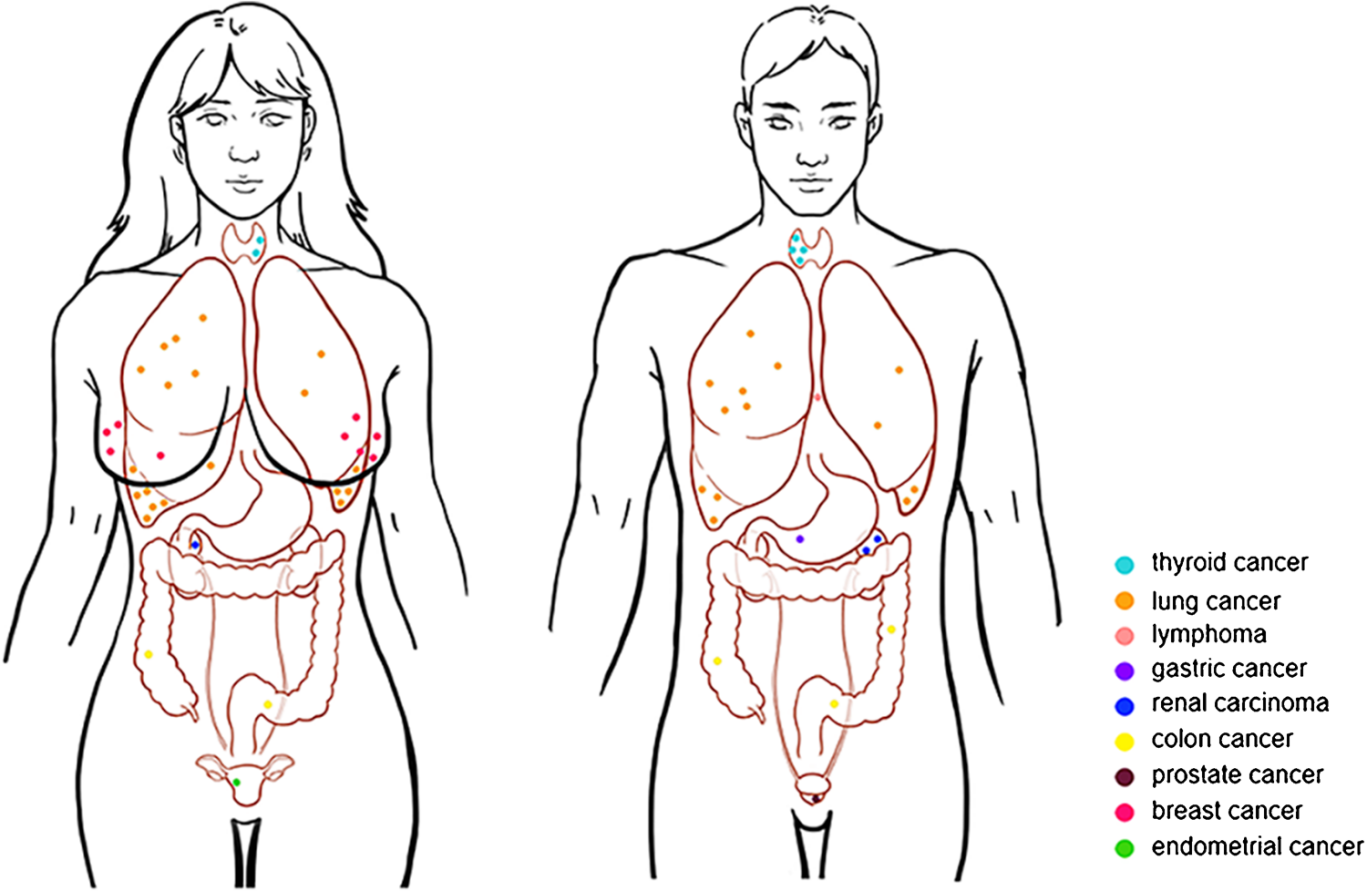
Distribution of cancers detect by PET/MRI combined with chest HRCT. Nine types of cancers were detected using PET/MR combined with chest CT, with various-colored dots representing distinctive types of cancers, and each dot representing 1 case. A total of 59 subjects were detected with cancers, including 25 males and 34 females

The distribution of cancers in male and female subjects is illustrated in Fig. 2. The types of cancers in these 59 subjects were as follows: lung cancer (*n* = 32, 54.2% of all cancers), breast cancer (*n* = 9, 15.2%), thyroid cancer (*n* = 6, 10.2%), colon cancer (*n* = 5, 8.5%), renal carcinomas (*n* = 3, 5.1%), prostate cancer (*n* = 1, 1.7%), gastric cancer (*n* = 1, 1.7%), endometrial cancer (*n* = 1, 1.7%), and lymphoma of the anterior mediastinum (*n* = 1, 1.7%). Fifty-four of the 59 subjects (91.5%) were at stage 0 or stage I, according to the 8th edition of the tumor-node-metastasis (TNM) staging system [16]. Twenty-seven of the 59 were non-lung cancers, accounting for 45.8%. Twelve of the 71 suggestive or possible subjects who had increased FDG uptake were later proved not to be cancers.

Eight (66.7%) of the 12 false-positive subjects were found to have abnormality in the thyroid gland (3 cases were benign evidenced by pathological biopsy, 5 cases with no positive findings during the follow-up), 2 cases (16.7%) in breast (1 case was breast fibroadenoma by puncture and 1 case was intraductal papilloma by surgery), 1 case (8.3%) in the nasopharynx (nasopharyngeal endoscopy proved to be inflammation), and 1 case (8.3%) in colon (the size was 2.6 × 2.4 cm and the pathology was tubular adenoma). Two subjects who had no abnormity in [^18^F]FDG PET/MRI scanning were histologically diagnosed with cancer in the follow-up, of which one female with breast cancer developed the symptom of a breast lump after 13 months from the examination and one male was detected with thyroid cancer after 3 months from the examination during the annual physical examination of his company. In total, 61 pathologic diagnoses of malignant tumors (2.0% of all subjects) were confirmed.

### Diagnostic performance of FDG PET/MRI combined with chest HRCT

**Fig. 3:**
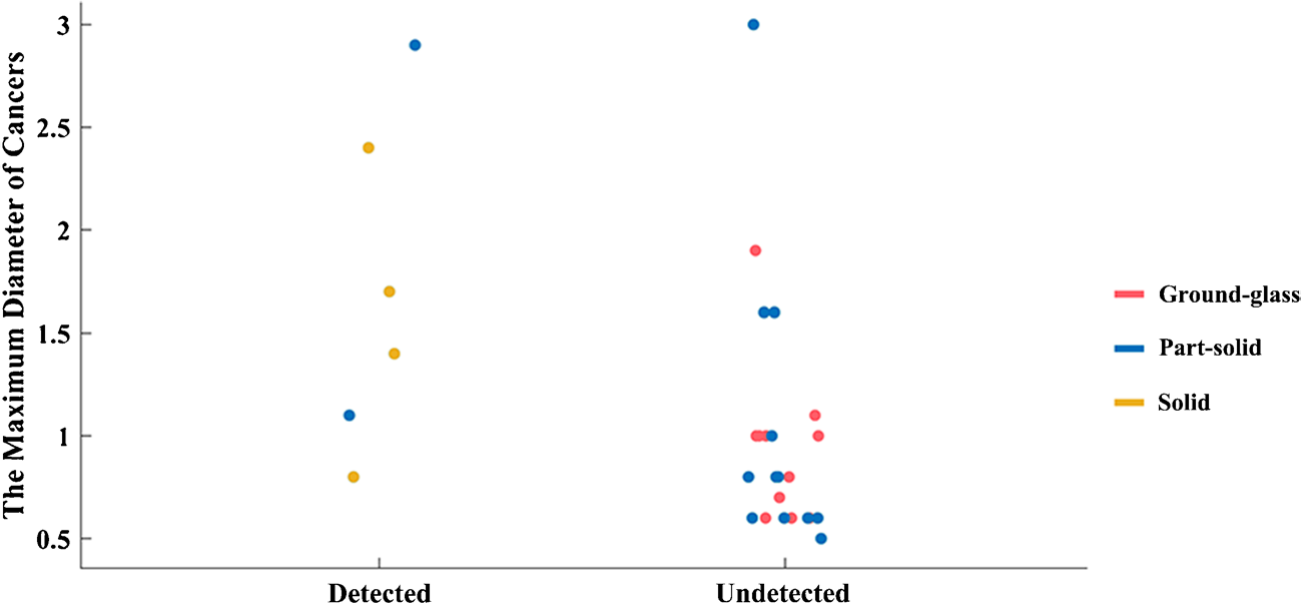
Types and sizes of the 32 lung cancers. Diagnostic performance of the PET/MR for solid (yellow dot), part-solid (blue dot), and ground-glass (red dot) lung cancers

In this study, 32 of the detected cancers (totally 61) were lung cancers, of which were at stage 0 or stage I. All of lung cancers could be found by chest HRCT, but only 6 (4 solid, 2 part-solid) of them could be detected by [^18^F]FDG PET/MRI due to mild or high FDG uptake. Twenty-six (ground-glass or part-solid) of 32 were missed in which FDG was not accumulated in the PET images. For PET/MRI, the maximum diameter of the long axis in the missed cancers was 3 cm and the minimum diameter of the long axis in the detection cancer was 0.8 cm (Fig. 3). Thus, chest HRCT greatly compensated the deficiency of [^18^F]FDG PET/MRI in detecting pulmonary lesions. Among the 29 malignant lesions outside lung, 27 cases were found to be suggestive or possible of malignant tumors by [^18^F]FDG PET/MRI, the remaining two were missed. In sum, 28 subjects (26 lung cancers and 2 non-lung cancers) considered to be normal or benign by PET/MRI alone were pathologically confirmed to be malignant; the true positive rate and false-negative rate of PET/MRI alone were 53.2% and 45.9%, respectively. PET/MRI combined with chest HRCT significantly increased the detection rate in whole-body cancer screening (*P* < 0.001); the true positive rate and false-negative rate of PET/MRI combined with chest HRCT were 95.1% and 3.2%, respectively.

The diagnostic performance of [^18^F]FDG PET/MRI combined with chest HRCT, [^18^F]FDG PET/MRI alone, and [^18^F]FDG PET/MRI alone for non-lung cancers are listed in Table 3. The cancer detection rate, sensitivity, specificity, PPV, and NPV for PET/MRI combined with chest HRCT were 2.0% (59/3020), 96.7% (59/61), 99.6% (2947/2959), 83.1% (59/71), and 99.9% (2947/2949), respectively. For PET/MRI alone, the metrics were 1.1% (33/3020), 54.1% (33/61), 99.6% (2947/2959), 73.3% (33/45), and 99.1% (2947/2975), respectively, and for PET/MRI alone in non-lung cancers, the metrics were 0.9% (27/3020), 93.1% (27/29), 99.6% (2979/2991), 69.2% (27/39), and 99.9% (2979/2981), respectively.

**Table 3:** Diagnostic performance of whole-body FDG PET/MRI in cancer detection

Imaging protocol	CDR	Sensitivity	Specificity	PPV	NPV
PET/MRI combined with CT	2.0%	96.7% (87.6–99.4)	99.6% (99.3–99.8)	83.1% (71.9–90.6)	99.9% (99.7–100.0)
PET/MRI alone	1.1%	54.1% (40.9–66.7)	99.6% (99.3–99.8)	73.3% (57.8–84.9)	99.1% (98.6–99.4)
PET/MRI alone for non-lung	0.9%	93.1% (75.8–98.8)	99.6% (99.3–99.8)	69.2% (52.3–82.5)	99.9% (99.7–100.0)

*CDR* cancer detection rate, *PPV* positive predictive value, *NPV* negative predictive value. Brackets indicate 95% confidence interval

## Discussion

In this study, we evaluated the ability of [^18^F]FDG PET/MRI combined with chest HRCT in whole-body early cancer detection. By combining [^18^F]FDG PET/MRI with chest HRCT, 59 of 3020 were correctly diagnosed with cancers (detection rate was 2.0%), and 91.5% of the detected cancers were at the early stages of cancers (stage 0 or stage I). This indicated that [^18^F]FDG PET/MRI combined with chest HRCT has the ability to detect early cancers in individuals without any history of the disease. To the best of our knowledge, this is the first study to evaluate the clinical values of [^18^F]FDG PET/MRI combined with chest HRCT for early cancer detection in a large cohort of asymptomatic subjects.

**Fig. 4:**
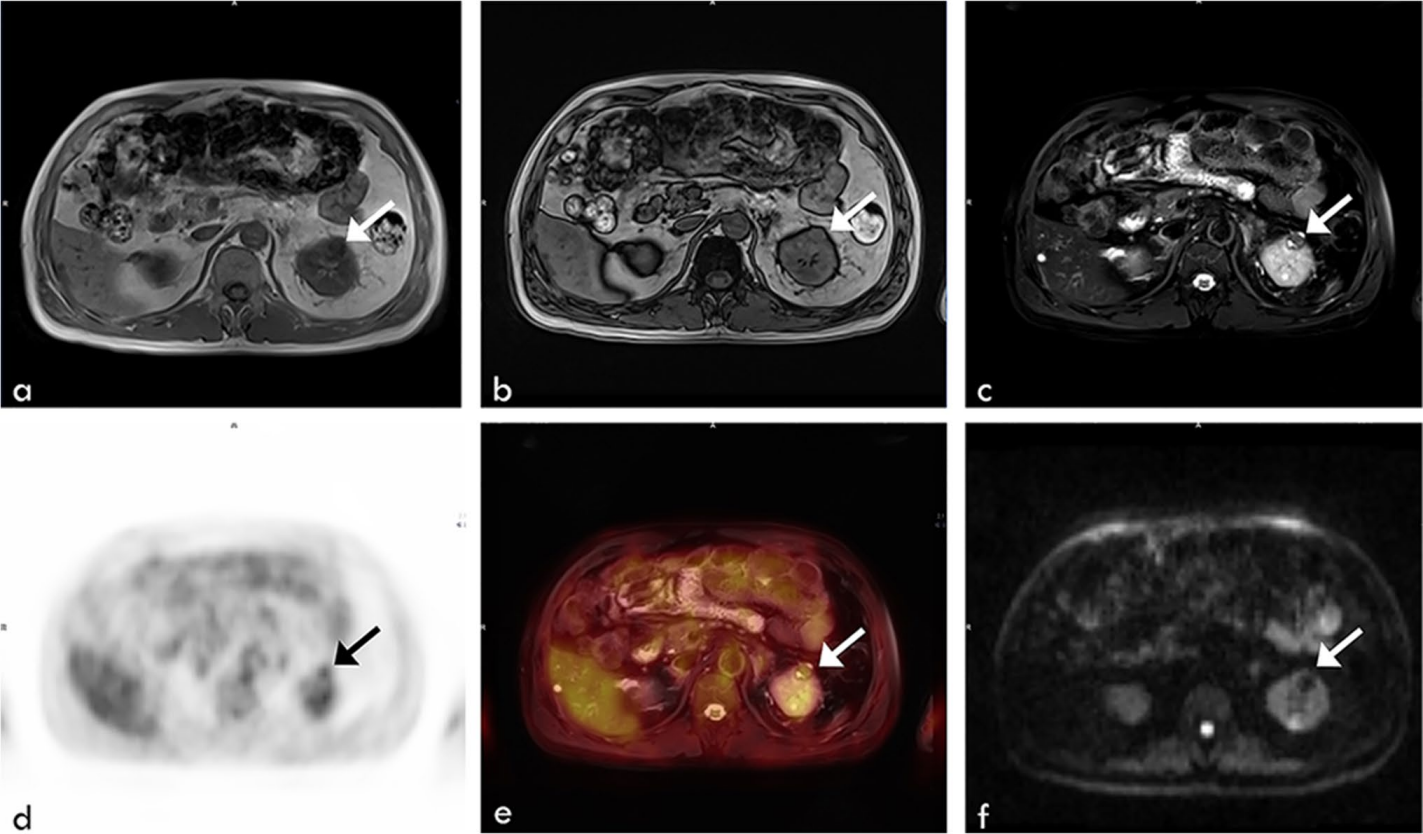
Axial PET/MRI images of a 65-year-old man with renal clear cell carcinoma. A lesion (arrow) was seen in the left kidney as an area of low signal intensity on the T1-weighted image (**a**, in phase; **b**, out of phase) and diffusion-weighted image (**f**), with cystic necrosis on the T2-weighted image (**c**). Mild FDG uptake was observed on the PET image (**d**) and on the fused T2-weighted and PET image (**e**)

Nine types of cancers were detected by [^18^F]FDG PET/MRI combined with chest HRCT in this study, including lung cancer, thyroid cancer, breast cancer, colon cancer, renal carcinomas, prostate cancer, gastric cancer, endometrial cancer, and lymphoma of anterior mediastinum, which were similar to previous studies [17–19]. Meanwhile, 91.5% of these cancers were at the early stages of cancers. The two high incidence cancers were lung cancer and breast cancer, which were consistent with global and Chinese cancer statistics [20]. It should be noted that three renal cancers were detected in this study with mild FDG accumulated on the PET imaging and the diagnosis was based on MRI morphological and signal changes (Fig. 4). In other words, renal cell carcinomas were missed in a study of 4881 asymptomatic subjects with whole-body [^18^F]FDG-PET scan [17]. Renal cancers generally display mild FDG accumulation, which is difficult to distinguish from physiologic accumulation. Compared with PET/CT, PET/MRI has advantages in detecting renal cell carcinoma [21].

**Fig. 5:**
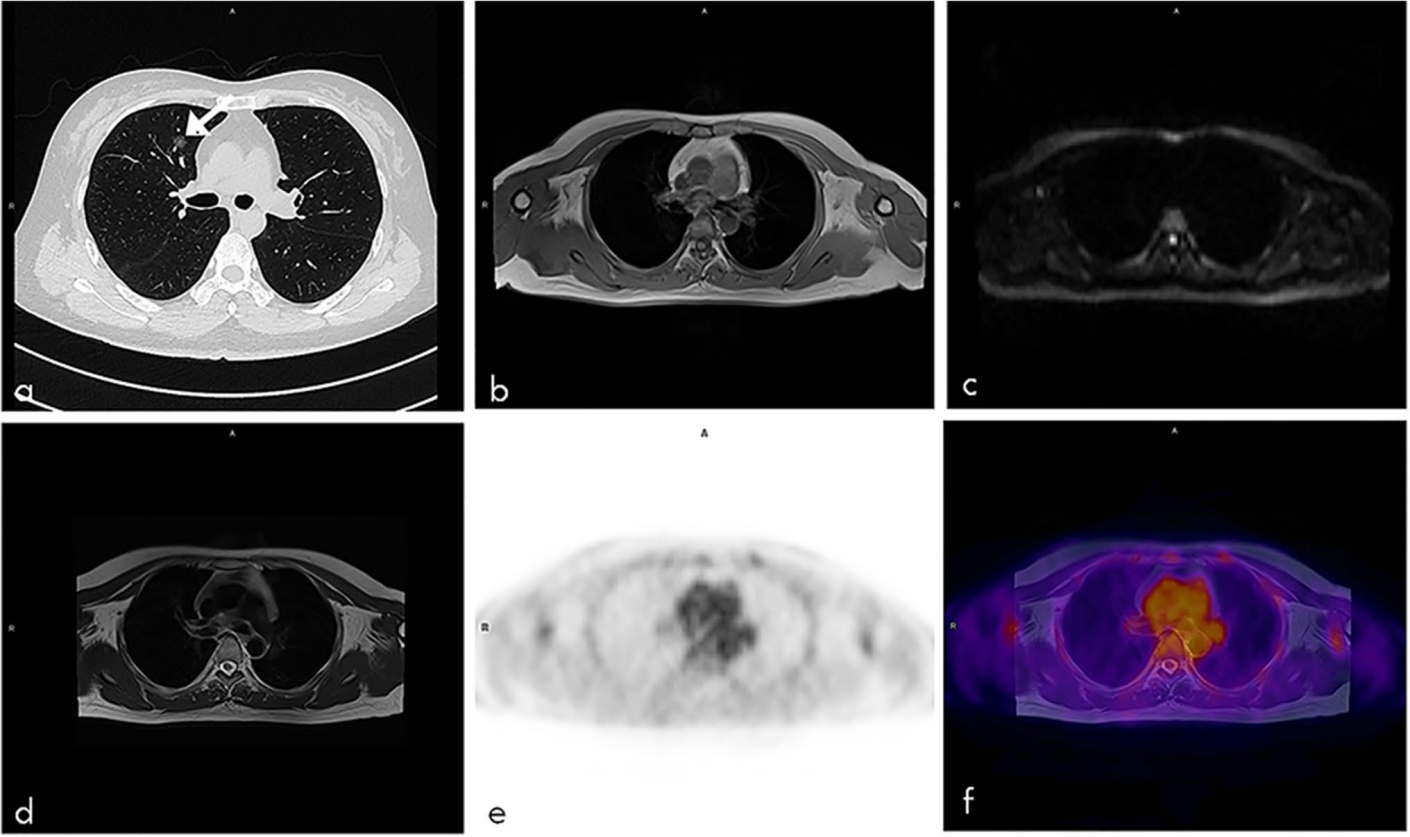
Axial CT and PET/MRI images of a 48-year-old woman with lung cancer. A ground-glass density nodule (arrow) is seen in the anterior upper lobe of the right lung with a size of 1.0 × 0.8 cm (**a**), which is negative on the T1-weighted image (**b**), diffusion-weighted image (**c**), T2-weighted image (**d**), PET (**e**), and fused T2-weighted and PET image (**f**). Pathology confirmed to be adenocarcinoma

The sensitivity of PET/MRI alone in this study was 54.1%, which was equivalent to the sensitivity of FDG PET in a retrospective study, but lower than that of PET/CT [22]. Obviously, PET/MRI alone had limited ability as a whole-body early cancer detection modality, mainly because the image quality of the lung MRI is usually inadequate for diagnosis and early-stage lung cancers may have no FDG uptake. Previous studies showed that the sensitivity of [^18^F]FDG PET/MRI in small lung nodules (< 5 mm) is less than [^18^F]FDG PET/CT [23]. However, another systematic review showed that [^18^F]FDG PET/MRI provided reasonably sensitive results in detecting malignant pulmonary lesions at the patient level compared with [^18^F]FDG PET/CT [24]. This might be attributed to different objective and different group of patients in these two reviews. For early lung cancer or small lung nodule detection, [^18^F]FDG PET/MRI is inferior to [^18^F]FDG PET/CT, while [^18^F]FDG PET/MRI is comparable even prior to PET/CT in lung cancer staging and restaging. It is worth noted that four of six detected lung cancers by PET/MRI were solid and 2 were part-solid. However, the 26 missed lung cancers by PET/MRI were ground-glass or part-solid, suggesting that PET/MRI is more suitable for the detection of solid lung cancers (Fig. 5). Besides, the ultrashort echo time (UTE) or high-resolution volumetric zero echo time (ZTE) sequence of MRI is reported to be more suitable for lung nodule detection [25]. Unfortunately, the UTE or ZTE sequence specific for lung nodule detection is not equipped on our PET/MRI. Previous studies have demonstrated that the simultaneous acquisition of radial VIBE and PET data with PET/MRI imaging has high sensitivity in detecting FDG-avid nodules and nodules 0.5 cm or larger [26]. Additionally, transverse T2BLADE images have shown the highest accuracy for lung nodule tumor staging, compared to other MR sequences on PET/MRI, including T2 HASTE, contrast-enhanced T1 FLASH, TrueFISP, non-enhanced T1 FLASH, and T1 3D Dixon VIBE [27]. Therefore, we utilized conventional VIBE, T2BLADE, and DWI sequences, supplemented by chest HRCT. If UTE or ZTE MRI sequences and parameters for lung cancers were selected, it could be helpful for detecting more pulmonary nodules [28–30]. When combined with chest CT, the sensitivity for whole-body cancer screening was significantly increased to 96.7%, which was much higher than that of [^18^F]FDG PET, PET/CT, and the combination of PET or PET/CT with multiple modalities (such as CT, MRI ultrasound of specific organ, tumor markers, and fecal occult blood testing, ranging from 50 to 85.19%) [17, 22, 31, 32]. This showed that chest HRCT is essential for detecting early cancer in asymptomatic subjects and the addition of HRCT could compensate the defect of PET/MRI in detecting lung nodules. Interestingly, our results showed the highest positive rate of early-stage lung cancer in asymptomatic subjects, which is consistent with the incidence of lung cancer in China [33]. Recently, the application of ultra-low-dose CT greatly facilitates the large-scale lung nodule detection due to its limited radiation compared with standard-dose CT [34]. Besides, it could reduce image noise and increase nodule detection by using deep-learning reconstruction [35]. Therefore, combined ultra-low-dose chest HRCT with PET/MRI could minimize the radiation dose, which might facilitate the clinical implementation of early cancer screening in a large group of asymptomatic individuals.

The 12 false-positive cases in this study included 11 benign tumors and 1 nasopharynx inflammation. Most of the 12 false-positive cases were located in the thyroid gland with high FDG uptake, accounting for 66.7% (8/12), which was consistent with previous studies that the malignancy rate of PET/CT in diagnosing FDG-avid thyroid incidentalomas (TIs) was 8–64% [36–40]. It should be noted that the pathology of a false-positive case in the colon was tubular adenoma, which was of clinical significance and required treatment with a size of 2.6 × 2.4 cm. Adding thyroid and breast ultrasound or mammography may be a preferable method for medical institutions to rule out the FP cases of thyroid and breast lesions.

False-negative cases in PET imaging can be classified into three types: very small size cases (< 10 mm), cell types with innately low FDG uptake, and cases located in the organs with high physiologic FDG uptake [41]. One false-negative case in this study was breast cancer, possibly due to the cell type of low FDG uptake. The other false-negative case was thyroid cancer, possibly due to both the small size and the cell type. According to a nationwide survey in Japan involving 50,000 subjects [19], stomach, prostate, and renal cancers were major limitations of PET and PET/CT, and the sensitivities were 27%, 45%, and 54%, respectively. However, no false-negative cases of these three cancers mentioned above were found in our study. For prostate and renal cancers, it could be explained that MRI anatomical images have high soft tissue resolution and DWI functional images are helpful to detect the lesions. Since 26 of 32 lung cancers were missed in detection with PET/MRI, it was important to combine FDG PET/MRI with an additional chest HRCT to reduce false negative. However, [^18^F]FDG PET/MRI has its specific advantages in whole-body cancer screening outside the lung. PET/MRI has a better resolution in soft tissue compared with PET/CT, especially for brain, breast, liver, pancreas, kidney, prostate, uterus, and lymph nodes. Therefore, PET/MRI could provide additional information for decision-making in contrast to conventional PET/CT. Besides, the estimated effective dose of PET/MRI (about 3.6 mSv) is reported to be lower than that of conventional PET/CT (about 17.6 mSv) in whole-body examinations [42]. It is worth noting that the appearance of full digital PET/CT (dPET/CT) changes this situation. dPET/CT greatly reduces effective dose of subjects by reducing radiopharmaceutical dose [43]. Unfortunately, it is lack of report about the dosimetric comparison between dPET/CT and PET/MRI combined with chest HRCT. According to the previous studies, the mean effective dose of dPET/CT excluding CT in patients (weight range from 38 to 100 kg) is 4.8 mSv [43]. The effective dose of CT component in PET/CT for whole-body scanning is depended on CT scanning parameters, which ranges from 2.9 to 7.2 mSv [44]. For chest HRCT, the effective dose is about 8 mSv [45]. Therefore, the effective dose of dPET/CT might be comparable or even lower than PET/MRI with incremental addition of diagnostic chest HRCT in whole-body cancer screening.

There are several limitations of our study. Firstly, this study had remarkable age- and sex-related biases: the subjects’ age distribution was mainly ranging from 40 to 50 years (70.7%), and only 15.2% subjects were over 60 years old. Thus, detection rate would be higher than 2.0% if the age bias was considered. Secondly, there could be few cancers missing by both PET/MRI and chest CT, especially through visual evaluation, which means that the detection rate, sensitivity, and PPV of this study might be lower than the true values. Thirdly, although it was longer than most studies, a longer follow-up can undoubtedly reveal more undetected cancers, thereby increasing the number of FN cases. In the next phase of our study, a longitudinal follow-up for up to 5 years and a prospective, multi-center survey are planned. Fourthly, the subjects underwent PET/MRI examinations at their own expense at a relatively high price. These subjects, who were relatively wealthy, usually had enough finances to guarantee well-being and undergo regular physical examinations. Therefore, we speculated that the cancer detection rate might be higher in other groups. Finally, MRI just provides structural information of lesions in this study, which lacks imaging-derived phenotypes of MRI images. It might be helpful to estimate the characteristics of lesions by making use of imaging-derived phenotypes [46].

## Conclusions

This study showed that [^18^F]FDG PET/MRI performs well in the early detection of non-lung cancers, but it is seemed insufficient for detecting early-stage lung cancers. Chest HRCT could be considered a powerful complementary modality for PET/MRI in whole-body early cancer detection.

### Supplementary information

ESM 1 (DOCX 20 kb)

## Data Availability

The data generated and analyzed in this study is available from the corresponding author on reasonable request.
