# Correction to: [^18^F]FDG PET/MRI combined with chest HRCT in early cancer detection: a retrospective study of 3020 asymptomatic subjects

**DOI:** 10.1007/s00259-023-06370-6

**Published:** 2023-08-23

**Authors:** Liling Peng, Yi Liao, Rui Zhou, Yan Zhong, Han Jiang, Jing Wang, Yu Fu, Le Xue, Xiaohui Zhang, Mingxiang Sun, Gang Feng, Zhaoting Meng, Sisi Peng, Xuexin He, Gaojun Teng, Xin Gao, Hong Zhang, Mei Tian

**Affiliations:** 1 Shanghai Universal Medical Imaging Diagnostic Center, Shanghai, China; 2 Department of Nuclear Medicine and PET-CT Center, The Second Affiliated Hospital of Zhejiang University School of Medicine, Hangzhou, Zhejiang China; 3 Key Laboratory of Medical Molecular Imaging of Zhejiang Province, Hangzhou, China; 4 Institute of Nuclear Medicine and Molecular Imaging of Zhejiang University, Hangzhou, China; 5 College of Information Science & Electronic Engineering, Zhejiang University, Hangzhou, China; 6 Human Phenome Institute, Fudan University, Shanghai, China; 7 Department of Oncology, Huashan Hospital, Fudan University, Shanghai, China; 8 Radiology Department, Zhongda Hospital Southeast University, Nanjing, Jiangsu China; 9 Key Laboratory for Biomedical Engineering of Ministry of Education, Zhejiang University, Hangzhou, Zhejiang China; 10 The College of Biomedical Engineering and Instrument Science, Zhejiang University, Hangzhou, Zhejiang China

**Correction to: European Journal of Nuclear Medicine and Molecular Imaging**

10.1007/s00259-023-06273-6

The authors regret that the “Methods” section of the abstract in the original article is incorrect. The correct “Methods” section appears below:

**Methods** This study included a total of 3020 asymptomatic subjects who underwent whole-body [^18^F]FDG PET/MRI and chest HRCT examinations. All subjects received a 2–4-year follow-up for cancer development. Cancer detection rate, sensitivity, specificity, positive predictive value (PPV), and negative predictive value (NPV) of the [^18^F]FDG PET/MRI with or without chest HRCT were calculated and analyzed.

The original article has been corrected.

The original article can be found at 10.1007/s00259-023-06273-6.

