# Supplementary material for: [18F]FDG PET/MRI combined with chest HRCT in early cancer detection: a retrospective study of 3020 asymptomatic subjects
Source: Eur J Nucl Med Mol Imaging. 2023 Jul 4;50(12):3723–34. doi: 10.1007/s00259-023-06273-6 (PMC10547651; doi:10.1007/s00259-023-06273-6)
Supplement: Supplementary file 1 — (DOCX 20 kb) [file 259_2023_6273_MOESM1_ESM.docx]

**Table S1 MRI sequences and technical details of the whole-body PET/MRI screening**

|  | **Region** | **Sequence** | **PAT** | **TR** | **TE** | **FOV** | **ST** | **VS** | **SG** |
| --- | --- | --- | --- | --- | --- | --- | --- | --- | --- |
| **Co-acquired with PET** | Whole body | Ax T1 VIBE | 2 | 4.47 | 1.96 | 440 | 3.1 | 1.4×1.4×3.1 | 1.2 |
|  | Whole body | Ax DWI  (b =50, 800 sec/mm2) | 2 | 3600 | 55 | 420 | 6 | 3.3×3.3×6 | 1.2 |
|  | Whole body | Dixon VIBE AC | 2 | 4 | 2.5 | 500 | 3.1 | 2.6×2.6×3.1 |  |
|  | Upper thigh/  Pelvis/  Upper abdomen | Ax T2 TSE BLADE FS | 3 | 2400 | 120 | 400 | 6 | 1.4×1.4×6 | 1.2 |
|  | Thorax | Ax T2 TSE BLADE | 3 | 2400 | 84 | 350 | 6 | 1.1×1.1×6 | 1.2 |
|  | Head-Neck | Ax T2 TSE BLADE | 2 | 4000 | 113 | 300 | 6 | 0.8×0.8×0.6 | 1.2 |
|  | Whole Spine | Sag T2 TSE DIXION | 2 | 3000 | 98 | 340 | 3.5 | 1.1×1.1×3.5 | 0.4 |
|  | Whole body | Cor T2 HASTE | 2 | 1000 | 91 | 380 | 7 | 1.2×1.2×7 | 2.8 |
| **Acquired after PET** | Head | Ax TIRM DARK FLUID | 2 | 8000 | 92 | 240 | 5.5 | 0.8×0.8×5.5 | 1.7 |
|  | Abdomen | Ax TI VIBE IN OPP | 2 | 4.44 | 1.34/2.57 | 380 | 3 | 1.2×1.2×3.0 | 1.2 |
|  | Pelvis  (female) | Sag T2 TSE | 2 | 5150 | 86 | 260 | 3 | 0.8×0.8×3.0 | 0.6 |

*PAT* parallel acquisition technique, *TR* repetition time in ms, *TE* echo time in ms, *FOV* field of view in mm, *ST* Slice thickness in mm, *VS* voxel size in mm, *SG* section gap in mm, *DWI* diffusion weighted imaging, *T1* T1-weighted, *T2* T2-weighted, *VIBE* volumetric interpolated breath-hold examination, *AC* attenuation correction, *HASTE* half-Fourier single-shot turbo spine echo, *STIR* short tau inversion recovery, *TIRM* turbo inversion recovery magnitude, *TSE* turbo spin echo, *FS* fat saturation, *IN* in phase, *OPP* out of phase, *AX* axial, *Cor* coronal, *Sag* sagittal.
